# Prediction and prevention of allergy and asthma in EAACI journals (2016)

**DOI:** 10.1186/s13601-017-0185-4

**Published:** 2017-12-02

**Authors:** Jean Bousquet, Clive Grattan, Thomas Bieber, Paolo Matricardi, Hans Uwe Simon, Ulrich Wahn, Antonella Muraro, Peter W. Hellings, Ioana Agache

**Affiliations:** 1 MACVIA-France, Contre les MAladies Chroniques pour un VIeillissement Actif en France European Innovation Partnership on Active and Healthy Ageing Reference Site, Montpellier, France; 2 INSERM U 1168, VIMA: Ageing and Chronic Diseases Epidemiological and Public Health Approaches, Villejuif, France; 3 UMR-S 1168, Université Versailles St-Quentin-en-Yvelines, Montigny le Bretonneux, France; 4 Euforea, Brussels, Belgium; 5 Dermatology Centre, Norfolk and Norwich University Hospital, Norwich, UK; 6 Department of Dermatology and Allergy, Rheinische Friedrich-Wilhelms-University Bonn, Bonn, Germany; 7 AG Molecular Allergology and Immunomodulation, Department of Pediatric Pneumology and Immunology, Charité Medical University, Berlin, Germany; 8 Institute of Pharmacology, University of Bern, Bern, Switzerland; 9 Pediatric Department, Charité, Berlin, Germany; 10 Food Allergy Referral Centre Veneto Region, Department of Women and Child Health, Padua General University Hospital, Padua, Italy; 11 Laboratory of Clinical Immunology, Department of Microbiology and Immunology, KU Leuven, Leuven, Belgium; 12 Faculty of Medicine, Transylvania University, Brasov, Romania; 13 CHU Montpellier, 371 Avenue du Doyen Gaston Giraud, 34295 Montpellier Cedex 5, France

**Keywords:** Allergy, Asthma, EAACI, Prediction, Prevention

## Abstract

The European Academy of Allergy and Clinical Immunology (EAACI) owns three journals: Allergy, Pediatric Allergy and Immunology and Clinical and Translational Allergy. One of the major goals of EAACI is to support health promotion in which prevention of allergy and asthma plays a critical role and to disseminate the knowledge of allergy to all stakeholders including the EAACI junior members.

The European Academy of Allergy and Clinical Immunology (EAACI) has three official journals: Allergy, Pediatric Allergy and Immunology and Clinical and Translational Allergy. One of the major goals of EAACI is to support health promotion in which prevention of allergy plays a critical role and to disseminate the knowledge of allergy to all stakeholders including the EAACI junior members [1].

The EAACI journals have reported on the prediction and primary and secondary prevention of allergic diseases and asthma in 2016. This paper summarises these achievements.

## Risk and protective factors

IgE-mediated allergy is much more common in Finnish compared with Russian Karelia, although these areas are geographically and genetically close. Many studies are trying to find the reasons explaining these differences. Higher concentrations of common environmental chemicals were measured in Russian compared with Finnish Karelian children and their mothers [2]. The chemicals did not explain the higher prevalence of atopy on the Finnish side.

Atopic dermatitis (AD) is a chronic inflammatory skin condition with a multifactorial pathogenesis. Several perinatal factors may influence the risk of AD. In a Danish nationwide register-based study [3], the risk of developing AD in the first 5 years of life was examined. Low birth weight and preterm birth were inversely associated with a lower risk of AD, while neonatal jaundice and birth during autumn or winter were associated with an increased risk of AD.

The prevalence of childhood AD varies considerably between ethnic groups. The Generation R Study assessed the role of environmental exposures and filaggrin (FLG) mutations on associations between ethnic origin and risk of childhood AD in 5082 children [4]. Compared with Dutch children, Cape Verdean, Dutch Antillean, Surinamese-Creole and Surinamese-Hindustani children had increased risks of AD in the first 4 years of life. Environmental and genetic risk factors partly weakened these associations.

Early gut colonization by *Bifidobacterium breve* and *B. catenulatum* differentially modulates AD risk in children at high risk of developing allergic disease [5]. Faecal samples were collected at age 1 week, 1 month and 3 months from 117 infants at high risk of allergic disease. Temporal variations in *Bifidobacterium* colonization patterns early in life are associated with later development of eczema and/or atopic sensitization in infants at high risk of allergic disease.

Evidence linking maternal psychosocial stress during pregnancy to subsequent child AD is growing, but the definition of AD is diverse and results are inconsistent. The first systematic review to date addressed prenatal maternal stress and the subsequent risk of atopy-related outcomes in the child [6]. Results suggest a relationship between maternal stress during pregnancy and atopic disorders in the child. However, the existing studies are of diverse quality and the wide definitions of often self-reported stress exposures imply a substantial risk for information bias and false-positive results.

Routine vaccinations can have non-targeted effects on susceptibility to infections and allergic disease. Such effects may depend on age at vaccination, and a delay in pertussis vaccination has been linked to reduced risk of allergic disease. In a population-based cohort of Melbourne, HealthNuts, 4433 12-month-old infants had skin tests and oral challenges to determine food allergy [7]. There was no overall association between delayed Diphtheria, Tetanus, Pertusis (DTaP) vaccination and food allergy; however, children with delayed DTaP had less AD and less use of AD medication. Timing of routine infant immunizations may affect susceptibility to allergic disease.

Body mass index (BMI) and physical activity in early childhood are inconsistently associated with atopic sensitization, AD and asthma in later childhood. Higher BMI and over or under physical activities in early childhood were associated with atopic sensitization, AD and asthma in later childhood [8]. Larger cohorts with repeated measurements of both predictors and outcomes are required to confirm the data.

Greater infant weight gain is associated with lower lung function and increased risk of childhood asthma. The role of early childhood peak growth patterns is unclear. A population-based prospective cohort study among 5364 children assessed repeated growth measurements between 0 and 3 years of age as well as BMI and age at adiposity peak [9]. Respiratory resistance and fractional exhaled nitric oxide were measured at 6 years of age. Greater peak height and weight velocities (PHV) were associated with lower respiratory resistance. Greater peak weight velocity (PWV) and BMI at adiposity peak were associated with increased risks of early and persistent wheezing. Childhood weight status partly explained these associations. No other associations were observed. Follow-up studies at older ages are needed to elucidate whether these effects persist at later ages.

The increased prevalence of atopic diseases has been largely studied in children and adolescents, but fewer data exist in adults. Results from the cross-sectional West Sweden Asthma Study in 30,000 randomly-selected individuals showed that there are different risk factor patterns for asthma, rhinitis and eczema in adults with some risk factors overlapping between these conditions. Allergic sensitization was a strong risk factor for current asthma and current rhinitis but not for current eczema. Obesity was a risk factor for current asthma and current rhinitis, while farm childhood decreased the risk for current asthma and rhinitis. Occupational exposure to gas dust or fumes and female sex was associated with an increased risk of current asthma and eczema [10].

Allergen exposure is associated with the development of allergic sensitization in childhood as reflected by global variations in sensitization patterns. However, there is little evidence to support a direct association. The Copenhagen Prospective Study on Asthma in Childhood 2000 birth cohort showed in children of 7 and 13 years that perinatal indoor aeroallergen exposure does not seem to affect development of allergic sensitization or rhinitis during childhood [11].

Finally, a hypothesis was proposed: may e-cigarette vaping boost the allergic epidemic by affecting human host defences, *Staphylococcus aureus* virulence and IgE sensitization? [12].

## Allergic March

Infants hospitalized for severe bronchiolitis are at increased risk of childhood asthma. A nested cohort study within the Massachusetts General Hospital Obstetric Maternal Study (MOMS) carried out a prospective cohort of pregnant women enrolled during 1998–2006 (n = 5407) [13]. AD was significantly associated with severe bronchiolitis in infancy. The mechanism of the AD-bronchiolitis association is unclear.

## Mechanisms

Finnish and Russian Karelian children have a highly contrasting occurrence of asthma and allergy: The methylation levels in the promoter region of the CD14 gene were higher in the Finnish compared to Russian Karelian children. However, the methylation variation of this candidate gene did not explain the asthma and allergy contrast between these two areas and the answer is not simple [14].

The role of FLG mutations during pregnancy and postpartum is unknown. FLG-genotyping was performed in a population-based sample of 1837 women interviewed in the 12th and 30th weeks of pregnancy and 6 months postpartum as part of the Danish National Birth Cohort study 1996–2002. Women with FLG mutations had an increased risk of AD flares during pregnancy and of enduring postpartum physical problems linked to perineal trauma during delivery [15].

First-born children are at higher risk of developing a range of immune-mediated diseases and may have a divergent activated T-cells profile suggesting in utero programming of the child’s immune system. In a subgroup of 28 children enrolled in the COPSAC2010 birth cohort, it was found that first-born infants display a reduced anti-inflammatory profile in Tv-cells at birth. This possible in utero ‘birth-order’ T-cells programming may contribute to a later development of immune-mediated diseases by increasing overall immune reactivity in first-born children as compared to younger siblings [16].

Although total IgE levels have been proposed as a biomarker for disease severity in AD and are increased in the majority of AD patients, they do not correlate with disease severity. During the synthesis of immunoglobulins, free light chains (Ig-FLCs) are produced in excess over heavy chains. In comparison with IgE molecules, Ig-FLCs have a very short serum half-life. Therefore, Ig-FLCs might be more suitable as a biomarker for disease severity during follow-up. However, immunoglobulin free light chains in adult AD patients do not correlate with disease severity [17].

## Epigenetics

DNA methylation in adulthood is associated with season of birth, supporting the hypothesis that DNA methylation could mechanistically underlie the effect of season of birth on allergy, although other mechanisms are also likely to be involved [18]. There may be an association between season of birth and blood DNA methylation in adulthood but a recent study was unable to replicate previous findings and the question is still open [19].

Early environmental factors are likely to contribute to CMA. In a small sample size from the Dutch EuroPrevall birth cohort study (N = 20 CMA, N = 23 controls, N = 10 tolerant boys), general hypermethylation was found in the CMA group compared to control children, while this effect was absent in the tolerant group [20]. Methylation differences were, among others, found in regions of DHX58, ZNF281, EIF42A and HTRA2 genes. Several of these genes are associated with allergic diseases.

Consumption of unboiled farm milk in early life prevents the development of atopic diseases. Milk is a complex signalling and epigenetic imprinting network that promotes stable FoxP3 expression and long-lasting Treg differentiation, crucial postnatal events preventing atopic and autoimmune diseases [21].

## Prediction

Profiles of allergic sensitization are poorly documented in infancy. Early polysensitization is associated with allergic multimorbidity in the Pollution and Asthma Risk: an Infant Study (PARIS) birth cohort of infants [22] as early as 18 months of age. Three profiles were found, differing in terms of allergic morbidity at 6 years. Early sensitization can predict allergic multimorbidity in childhood, and in the case of early polysensitization, multimorbidity is more frequent as early as infancy [23].

The longitudinal pattern of allergen-specific IgE levels from the prenatal stage to early life has remained largely unexplored. 103 mother-infant pairs, part of an ongoing population-based prospective birth cohort study in Taiwan, found that an influence of maternal allergen-specific IgE levels on infant immune response might occur at birth and then wane in infants at 12 months of age [24].

A longitudinal study of maternal body mass index, gestational weight gain, and offspring asthma was carried out in the Growing Up Today Study [25]. Physician-diagnosed asthma during childhood or adolescence was reported by 2694 children (21%). Maternal prepregnancy overweightness and obesity were associated with offspring asthma. The relation of several prenatal factors to risk of childhood asthma supports the early origins hypothesis for asthma.

## Prevention

Breastfeeding is associated with a lower risk of asthma symptoms in early childhood, but its effect at older ages remains unclear. The Food Allergy and Intolerance Research (FAIR) cohort (n = 988) [26] showed inconsistent protective effects of nonexclusive and exclusive breastfeeding against long-term allergic outcomes. The Generation R Study [27] examined the associations of duration and exclusiveness of breastfeeding with asthma outcomes in children aged 6 years, and whether these associations were explained by atopic or infectious mechanisms. Breastfeeding patterns may influence wheezing and asthma in childhood, which seem to be partly explained by infectious mechanisms.

Prevention guidelines for infants at high risk of allergic disease recommend hydrolysed formula if formula is introduced before 6 months, but evidence is mixed. Adding specific oligosaccharides may improve outcomes. A partially hydrolysed whey formula containing oligosaccharides does not prevent AD in the first year in high-risk infants [28]. The immunological changes (increased regulatory T-cell and plasmacytoid dendritic cell percentages) that were found require confirmation in a separate cohort.

Data on the long-term impact of hydrolyzed formulas on allergies are scarce. The GINI (German Infant Study on the influence of Nutrition Intervention) trial participants (n = 2252) received one of four formulas in the first four months of life as breast milk substitute if necessary: partial or extensive whey hydrolyzate (pHF-W, eHF-W), extensive casein hydrolyzate (eHF-C) or standard cow’s milk formula (CMF) as Ref. [29]. Between 11 and 15 years, the prevalence of asthma and the cumulative incidence of AR were reduced in the eHF-C. The cumulative incidence of AD was reduced in pHF-W and eHF-C, AD prevalence was reduced in eHF-C. No significant effects were found in the eHF-W group on any manifestation, nor was there an effect on sensitization with any formula. In high-risk children, early intervention using different hydrolyzed formulas has variable preventative effects on asthma, allergic rhinitis and AD up to adolescence.

Nutritional adequacy of a cow′s milk exclusion diet in infancy is essential since infants with suspected cow’s milk allergy are required to follow a strict milk exclusion diet [30]. In a group of UK infants (subgroup of the Prevalence of Infant Food Allergy study), the diets of 39 infants (13 milk-free and 26 controls) were assessed. Although infants consuming a milk-free diet have a nutritional intake that is significantly different to matched controls who are eating an unrestricted diet, this difference is not constant and it is not seen for all nutrients.

The impact of the elimination diet on growth and nutrient intake in children with food protein induced gastrointestinal allergies was examined in children with delayed type allergies [31]. A prospective, observational study was performed at a tertiary gastroenterology department in children ranging in age from 4 weeks to 16 years. With appropriate dietary advice, including optimal energy and protein intake, hypoallergenic formulas and vitamins and mineral supplementation, growth parameters increased from before to after dietary elimination. These factors were positively associated with growth, irrespective of the type of elimination diet and the numbers of foods eliminated.

The prevalence of food hypersensitivity in the UK is still largely open to debate [32]. In a population based birth cohort study conducted in Hampshire, UK as part of the European Initiative on Food Allergy, EuroPrevall, birth cohort study, 1140 infants were recruited with 823 being followed up until 2 years of age. The diagnosis of food allergy was ascertained by positive double-blind, placebo-controlled food challenge (DBPCFC). Cumulative incidence of food hypersensitivity by 2 years of age was 5.0%. The cumulative incidence for individual food allergens were hens’ egg 2.7% (1.6–3.8); cows’ milk 2.4% (1.4–3.5); peanut 0.7% (0.1–1.3); soy 0.4% (0.0–0.8); wheat 0.2% (0.0–0.5) and 0.1% (0.0–0.32) for fish. Just under half the infants with confirmed food hypersensitivity had no demonstrable IgE.

In the Probiotics in Prevention of Allergy among Children in Trondheim (ProPACT, n = 259) study, AD prevention in children following maternal probiotic supplementation does not appear to be mediated by breast milk TSLP or TGF-beta [33].

## The political agenda

Preventive strategies for allergic diseases need to be anchored on a strong political agenda to implement the results of the research into practice. The European Symposium on Precision Medicine in Allergy and Airways Diseases at the European Union Parliament (October 14, 2015) stressed that the socioeconomic impact of allergies and chronic airways diseases cannot be underestimated [34]. Participants underscored the need for optimal patient care in Europe, supporting joint action plans for disease prevention, patient empowerment, and cost-effective treatment strategies. AIRWAYS-ICPs (Integrated care pathways for airway diseases, Action Plan B3 of the European Innovation Partnership on Active and Healthy Ageing, DG Santé and DG Connect) focuses on the prevention and integrated care of chronic diseases. It has proposed a scale-up strategy for the management and prevention of allergic diseases using the recommendations of the European Innovation Partnership on Active and Healthy Ageing [35]. ARIA, the Allergic Rhinitis and its Impact on Asthma (ARIA) initiative commenced during a World Health Organization workshop in 1999, is also targeting preventive strategies to prevent asthma and rhinitis by implementing emerging technologies using the ARIA Allergy Diary app and the ARIA allergy companion app [36]. In close collaboration with the European Forum for Research and Education in Allergy and Airways diseases (EUFOREA) [37], an action plan for increasing awareness on prevention, patient empowerment and cost-effective treatments is being elaborated.

## Abbreviations

AD: atopic dermatitis
AR: allergic rhinitis
BMI: body mass index
CMA: cow’s milk allergy
CMF: standard cow’s milk formula
DTaP: diphtheria, tetanus, pertusis
EAACI: European Academy of Allergy and Clinical Immunology
eHF-C: extensive casein hydrolyzate
eHF-W: extensive whey hydrolyzate
FLG: fillagrin
FoxP3: forkhead box P3
Ig-FLCs: immunoglobulin, free light chains
LC-PUFAs: long chain polyunsaturated fatty acids
pHF-W: partial whey hydrolyzate
PHV: peak height and weight velocities
PWV: peak weight velocity
SNIP: single nucleotide polymorphisms
TGF-β: transforming growth factor-β
Treg: T regulatory cell
TSLP: thymic stromal lymphopoietin
